# Outlines of Comparative Anatomy

**Published:** 1835-07-01

**Authors:** 


*Outlines of Comparative Anatomy.* By ROBERT E. GRANT, M.D., &c. Professer of Comparative Anatomy and Zoology in the University of London, &c. Part I. containing *Osteology, Ligaments, and Muscles; illustrated with sixty-five Woodcuts.* London, 1835. 8vo. pp. 144.

THIS work is strictly what its title denotes,—Outlines of Comparative Anatomy.

In the part now before us, the general structure of the bones, ligaments, and muscles, is traced through the various grades of animated being up to man; the anatomical facts are accurately stated, and no physiological theories are deduced from them; hence we have nothing to dispute, and little to expatiate upon; a hard and hapless situation for reviewers.

In attributing a skeleton to all animals, we are correct only in as far as the osseous system is considered as that of *support*, without reference to its texture, or its position in relation to other parts: of the various offices to which it is subservient in different classes, that of supporting the frame is alone common to it in all. Thus, in the higher animals, the skeleton affords a great system of levers for muscular action, and forms a defence only for particular organs; in the *testacea*, while it affords attachment to muscles, it is of still more importance as a general defence for the soft parts: again, in animals whose habits or mode of existence are peculiar, we sometimes find it ministering to such peculiarities; thus, in birds, it is a pneumatic apparatus, adapted to their necessities as travellers of the air; in many quadrupeds, the skull is an offensive weapon used in contending with their adversaries; in the *pholas*, the shell is a tool, wherewith the animal excavates, in the solid rock, a cavern for its dwelling, and, by some process imperfectly known, enlarges its habitation, as its own bulk increases; but, in all classes, the osseous system affords the means of support and connexion to the frame, and, as no animal is destitute of some such support, a skeleton, or something analogous to it, in its most invariable and important function, may be said to pervade the animal creation.

Anatomically considered, the organs of support vary infinitely in their structure and distribution, in the lower animals, sometimes bearing an analogy to the bones, at others to the skin; but in all consisting of some texture firmer than the rest, which serves to determine the size and shape of the entire organism, and to maintain its integrity against the external impressions to which, under ordinary circumstances, it is subjected.

"These denser parts of the body serve as a solid framework to give form and solidity to the whole fabric, and to protect the more delicate organs. They consist for the most part of earthy materials separated from the food by the vital processes of the animal, and may be placed on the exterior or in the interior of the soft parts. These inert materials, or passive organs of locomotion receive their forms from those of the soft parts, and are liable to change with the varying conditions of the contiguous living parts. When placed on the exterior of the body, they may, without being organized, keep pace with the progress of growth in the living parts, by being periodically cast off and renewed; or they may increase by the addition of more extended layers to their surface; or their dimensions may be continually influenced by the contact of the parts which formed them. But when this solid framework is internal, and is everywhere surrounded by the soft parts, giving attachment to muscles, or enveloping and protecting delicate organs, it cannot be conveniently removed from the system in a mass, nor preserve its proportions by the mechanical addition of layers to its surface, and is generally organized or permeated in every point by the soft parts which absorb the decayed materials and renew them particle by particle. The earthy materials thus formed by animals for the support of their soft parts are various, and their particles are generally united together by means of a condensed albuminous or gelatinous matter, which gives firmness and tenacity to the mass. Silica is found in the lowest forms of radiated animals; carbonate of lime in the molluscous classes; carbonate and phosphate of lime in the articulated animals, and phosphate of lime in the organized skeletons of the vertebrata. These earths, in consolidating, assume forms by the influence of laws which are in accordance with their ordinary physical properties, this we observe most obviously in the lowest animals, and least in the highest classes where the crystalline arrangement of the particles is most equivocal; but under every condition they alike form a normal part of the structure, a solid framework more or less complete, constant in its form and structure in the same species, and varying in its form with the specific differences of animals. This solid framework forms the osseous system of animals, or the skeleton, as it has been termed from the dry and earthy nature of the materials which compose it. The osseous system, though not the most important nor the most universal system of animal organization, is met with under some form in every class of the animal kingdom, though not in all the animals of each class." (P. 1.)

We shall here briefly allude to the organs of support, in some of their less familiar forms, as developed in animals low in the scale of beings.

*Organs of Support in the Radiated, or Cyclo-Neurose Classes.*

In this lowest division of the animal kingdom, the skeleton is extremely various, consisting of one solid mass, or of parts symmetrically arranged; composed of a flexible horny substance, of siliceous or calcareous spicula, or of masses of carbonate, with some phosphate of lime. In these animals, the osseous parts appear to be extra-vascular, and to increase by the simple apposition of new portions.

I. *Polygastrica.* In this class the skeleton exists,

*a.* As a mere condensation of the common integument; as in *volvox globator,* so abundant in stagnant pools.

"This spherical transparent green coloured, tuberculated animalcule exhibits in its interior numerous smaller, round, spotted, and similarly formed beings moving to and fro, and the entire volvox does not change or vary its external form while it is seen swimming about slowly with the enclosed young. When the exterior capsule, or the parent animalcule bursts, and the young have escaped, we observe its fragments to retain their original form with some degree of elasticity when they are tossed about in the fluid by the motions of other animalcules." (P. 3.)

*b.* As a thin elastic sheath enveloping the body, into which the animal can withdraw itself, and from which it can protrude its anterior ciliated portion for the purposes of nutrition, respiration, or locomotion; as in *vaginicola innata,* a common marine animalcule.

II. *Poriphera.*—The skeleton of poripherous animals consists of separate minute, earthy, crystalline spicula, connected together, by a condensed, elastic, cellular substance; or of tubular elastic filaments of a horny consistence. These hard parts are developed internally throughout the whole cellular tissue of the body, and are often protruded externally through the surface, to protect the pores, or the large vents. The earthy spicula in most of these animals are silicious, in many they are calcareous, and, like the horny filaments of other species, they appear to be tubular like many natural crystals, and to have no aperture leading into their internal cavity. The spicula are generally united into fasciculi by an enveloping glutinous, or condensed cellular substance, and by the junction of these fasciculi in various modes, fibres are formed which traverse every part of the body, forming the boundaries of canals and orifices, and giving form and support to the whole of the gelatinous or soft cellular substance of the animal. The forms of these hard parts are different in every distinct species of these animals, and they are constant in the same, so that they present useful characters for the distinction of species in this polymorphous class. They are formed from materials due to the vital energies of the animal, and they form normal and necessary parts of its structure, like the solid skeletons of higher animals." (P. 5.)

The skeleton, composed of calcareous, is generally more complex than that formed of siliceous spicula, and these two kinds of spicula do not appear to occur in the same animal. Siliceous spicula, similar to those of the poripherous animals, occur abundantly in the plants to which such animals are most nearly allied. *Haliclona occulata* affords an example of the siliceous, and *Leuconia compressa* of the calcareous skeleton.

In the skeleton composed of horny spicula, fine, elastic, tubular filaments are united together, and distributed around the pores, canals, and vents. Sometimes the cavity of the filaments is filled with an opake matter, which renders them more brittle; but they generally contain only a colourless fluid, as in *Spongia officinalis,* which may be taken as an example of the horny skeleton.

II. *Polypiphera.*—"The skeletons of zoophytes present a great variety of forms and characters, being branched or globular, or filiform, free or fixed, solid, massive, and calcareous, or soft, flexible, and horny, external or internal. The animals of this class obtaining their food by polypi, or highly organized sacs developed from the fleshy substance of the body, we generally find the skeletons, whether external or internal, to present cavities or cells for the reception and protection of these delicate organs; and the various forms of these cells constitute a principal distinction among the skeletons of this class. The simplest forms of the skeleton are presented by the horny zoophytes, or keratophytes, where it sometimes consists of tough, soft, flexible filaments, which surround the cells of the polypi throughout the whole mass of the body, as in the *alcyonium* and *lobularia.* These form a transition from the horny species of poriphera to the more distinct forms of keratophytes. In the horny species of zoophytes the skeleton sometimes forms a tubular external sheath enveloping the fleshy substance throughout all the ramifications of the body, as in all the *sertulariæ, plumulariæ, antennulariæ,* and many other soft, flexible, and ramified forms. The horny skeleton is sometimes formed by the deposition of successive layers within the fleshy substance of the animal, as in the *gorgonia* and *antipathes.* We have an example of an external, tubular, horny skeleton in the common *campanularia dichotoma,* where we observe it enveloping as a sheath the fleshy substance which occupies the centre of all the divisions of the root, the stem, and the branches. The exterior horny sheath which is exuded upon the surface of the flesh expands at the extremities of all the branches to form cells, for the lodgment of polypi. The base of attachment, spread out and ramified like a root, exhibits the same fleshy interior, and the horny covering extended over all its divisions. In the axillæ of many of the branches we observe large vesicles, for the protection and development of the embryo. These vesicles in the vaginiform keratophytes are composed of the same firm pellucid substance as the rest of the skeleton; and from the constancy of their forms in the same animal, and their differences according to the species, they afford useful characters for the distinction of these animals. They are deciduous parts of the skeleton, as they fall off after the matured gemmules have escaped from their interior. These gemmules are seen in little ciliated capsules, and the polypi are extended in various attitudes from the cells, in search of animalcules as food. The skeleton of these vaginiform zoophytes often presents a jointed appearance on the stem or branches, as in the *campanularia;* these consist of circular indentations of the surface which do not pass through the interior of the body, where they would interrupt the circulation of the nutritious fluid which passes through the fleshy substance in all parts of the body. They allow of a certain degree of flexibility at the most suitable parts of the skeleton, and in some of the horny *cellariæ* they are connected with the deciduous character of the branches. In the *gorgonia,* and some other cortical zoophytes, there is an exterior fleshy substance in the living state which covers all parts of the horny skeleton. This fleshy exterior crust is indeed the animal, which forms by the deposition of successive layers the whole of the flexible, branched, horny, and solid internal skeleton. If we make a transverse section of a thick portion of the *gorgonia,* or *antipathes,* we can easily perceive the concentric layers of which it is composed; and by peeling off the cortical fleshy mass from the exterior, and placing this living flesh in the sea, we find it to secrete a new internal horny axis for itself. The polypi, which are always and necessarily continuations of the fleshy substance of zoophytes, are developed from this thick fleshy crust in the cortical kinds, and hence we do not see any appearance of cells on the central horny axis in these animals, after the flesh has been removed.

"In the calcareous zoophytes the solid mass forming the skeleton is composed chiefly of the carbonate of lime, with a little of the phosphate; and the same condensed glutinous matter which forms the entire skeleton in the keratophytes, is diffused through the whole of the calcareous mass in the more solid lithophytes, where it serves to agglutinate the earthy particles, and to give solidity and tenacity to the entire mass. The calcareous skeletons of lithophytes are for the most part internal, massive, and consisting of a single piece. In madrepores, and many similar forms, the thin fleshy crust penetrates to a considerable extent the loose, porous surface of the calcareous mass, from which it is capable of receiving some protection, and consequently we perceive distinct indications of the positions of the polypi on the surface of the skeleton in these animals. These cells, for the protection of the polypi, have generally a radiated lamellar structure, and vary remarkably in their size and also in their form in different lithophytes. They are very minute in the *porites,* larger in the *madrepores,* still larger in the *caryophylliæ,* and the *fungia agariciformis* forms but one enormous cell for the lodgment of a polypus like an actinia.

"In some of the lithophytes the fleshy crust, as in the cortical kinds of keratophytes, is of great thickness, and the polypi developed from this fleshy exterior mass leave no indications of their position on the surface of the internal calcareous axis. This is seen in the common red coral, which is a solid internal calcareous skeleton, striated with superficial longitudinal grooves, but presenting no calcareous cells for the polypi, which are protected solely by the fleshy thick covering of which they form parts. In the *agariciæ*, *meandrinæ*, and many others, we observe a laminated general surface of the skeleton for the protection of the fleshy mass, but no distinct cells for the polypi. In the *virgulariæ* the skeleton consists of a straight internal calcareous solid cylindrical pillar, occupying the longitudinal axis of the body, and protruding from the lower part of the animal. In the pennatulæ the internal calcareous axis is soft and flexible at its extremities, from the abundant proportion of glutinous matter in its composition, and to allow of the necessary contractions and extensions of the animal's body in a longitudinal direction. In the *isis* the internal solid calcareous skeleton is jointed at regular and short distances throughout the whole body, and there are no external cells for the polypi, which are entirely confined to the thick fleshy crust which covers the entire animal in the living state. The joints here consist of the same glutinous tough matter which pervades the whole calcareous axis, and are only uncalcified portions of the general solid axis. They are formed by concentric layers, like the calcified solid portions of the skeleton, and they allow of considerable flexion in the branches and stem of this delicate, ramified, and highly organized animal. As in most other classes of invertebrata, we find many zoophytes which are destitute of an external or internal skeleton, as the common fresh-water polype, or *hydra*. Besides the solid internal skeleton in the corticiferous zoophytes, we commonly find in the fleshy crust itself minute calcareous spicula. These small spicula compose the hard crust which is seen covering the horny axis of the *gorgonia*, as it is commonly preserved dried in cabinets; and in their occurring thus spread through the general fleshy mass in *gorgoniæ*, *lobulariæ*, and many other zoophytes, we observe a lingering analogy with the spicular form of the skeleton in the class of poripherous animals, especially in the calcareous group. The skeleton in the keratophytes is exuded from the fleshy substance in a soft and semi-fluid state, and quickly hardens after its separation from the living parts upon which it is moulded." (P. 9.)

"IV. *Acalepha.*—Although there are no solid skeletons in any of the soft, gelatinous, free, and floating animals of this class, we generally perceive some firmer cartilaginous portions of the body which afford support to the organs of progressive motion or of prehension. There are crescentic cartilaginous laminæ around the inferior central part of the body in the medusæ which give support to the contracting fleshy overhanging mantle, and to the absorbent tubes prolonged from that part. There are firm superficial longitudinal bands in most of the ciliograde acalepha for the support of those minute vibratile fins, by the motions of which they are carried through the sea. From the feebleness of their muscular system, and from their swimming habits, it is obvious that the acalepha can only support the lightest forms of the skeleton.

"In the *velella limbosa*, which floats on the surface of the sea, there is a thin flexible perpendicular crest, which is covered with a thin layer of the deep blue coloured mantle, and which rests obliquely on a horizontal stronger transparent flexible plate. The thin perpendicular crest, which rises above the water and serves as a sail, appears to be composed of the same condensed glutinous or horny substance which composes the skeletons of the keratophytes. The horizontal plate is thicker, concave below, marked with concentric lines of growth, and gives support to the deep blue mantle above, to the delicate marginal tentacula, to the numerous tubular suckers, and to the stomach placed beneath this concave horizontal plate.

"The *porpita*, which is another of these floating *acalepha*, presents a similar thin plate, to this horizontal lamina of the velella, for the support of the same parts, but of a round form, of a white colour, and of a porous texture. These two simple genera of acalepha present examples of the thin, light, and delicate forms of the skeletons which we find in almost all the floating marine invertebrata." (P. 15.)

In the next higher class, the *Echinoderma*, the skeleton begins to be more defined, and, through the succeeding classes, is gradually developed into forms more generally familiar to anatomists: we shall not, therefore, extend our extracts from the osteological portion of the work, the few we have given being intended merely as examples of the author's manner of treating his subject. The myology only commences near the conclusion of this first part, and breaks off in the abrupt style now in fashion; against which, and the whole system of publishing books in *frustules*, we beg leave here to enter our formal protest: it is unsatisfactory, inconvenient, and expensive, and can be approved of only by lazy and desultory readers.

The following account of the vibratile cilia, which are at once organs of locomotion and respiration in some of the radiated animals, shows how complex and perfect are the muscular actions in animals very low in the scale of being, and sufficiently demonstrates the futility of that hypothesis which regards nature as a tyro toiling through the difficult work of creation in a long series of experimental lessons: how can this be the case when we find, in the cilia of a polypus, such precision and activity of motion, that the important function of respiration is left, as it were, to be sustained by the vigilance and dexterity of the animal itself!

"In the vaginiform zoophytes the internal currents of the body are not produced by the contractions of the internal fleshy parietes, though compared by Cavolini to a heart, but by the action of vibratile cilia; and the open base of the polypi often contracts, as if by a movement of deglutition. The polypi being the organs by which the food is attracted, seized, and digested, and the surface of the body aerated, they possess the most complicated structure and the highest vitality. The tentacula surrounding the mouth, as in the *flustra carbesia*, have generally minute vibratile cilia disposed along their sides, by the action of which currents of water, conveying animalcules and other food, are directed towards these prehensile and digestive cavities. In these complicated and highly irritable polypi of the *flustra*, we perceive numerous muscular bands, connecting the lower part of the polypus with the aperture of the cell; and others passing from the same part of the polypus downwards, to connect it with the bottom of the cell. The vibratile cilia move the currents outwards along one side of each tentaculum, and inwards along the other side, and they are generally visible only while they move in the direction of the currents which they produce, that is, in their forward stroke. When in full activity the cilia are invisible in their backward movement, from the velocity with which they resume their position, to commence a new stroke forward. And as vibratile cilia are thus generally perceptible only in their slow forward impelling movement, they have some resemblance to a stream of globules flowing always in one direction. There are about fifty cilia on each side of a tentaculum in the *flustra carbesia*, and nearly forty millions on a moderate specimen of the entire animal. In many zoophytes they are much more numerous; so that these fixed and plant-like animals, though possessing a very low degree of irritability in their general mass, are well provided with active organs to bring their food towards them, and to renew the stratum of water in contact with their surface, for the purpose of respiration." (P. 133.)

We conclude a notice, rather than a review, of this accurate and well-digested work, by recommending it strongly to the perusal of all students of medicine—we say of all students of *medicine*; for every physician must be a physiologist, and no man can be a physiologist without a competent knowledge of comparative anatomy.

